# Authors’ reply

**Published:** 2010

**Authors:** Yoshinori Mitamura, Masayasu Kitahashi, Mariko Kubota-Taniai, Shuichi Yamamoto

**Affiliations:** 1 Department of Ophthalmology and Visual Science, Chiba University Graduate School of Medicine, 1-8-1 Inohana, Chuo-ku, Chiba 260-8670, Japan; 2 Department of Ophthalmology, Institute of Health Biosciences, The University of Tokushima Graduate School, 3-18-15 Kuramoto, Tokushima 770-8503, Japan

Dear Editor,

We appreciate the comments by Chhablani[1] regarding our article.[2] The best treatment for polypoidal choroidal vasculopathy (PCV) has still not been established. Our results suggest that photodynamic therapy (PDT) may be more effective than intravitreal bevacizumab (IVB) shortly after treatment for PCV. However, we did not evaluate the efficacy of intravitreal ranibizumab (IVR). Ranibizumab is a smaller molecule than bevacizumab, and the penetration of ranibizumab into the subretinal pigment epithelium space might be better than that of bevacizumab. As mentioned in our article, further studies to evaluate the efficacy of other anti-vascular endothelial growth factor (VEGF) agents and combination therapy of PDT and anti-VEGF agents are required and ongoing.

In reply to the first comment “it might be difficult to treat multiple widely distributed lesions with a single beam of PDT,” we usually treat polyps in and around the macula with a single beam of PDT and other remote lesions are not treated or treated using direct photocoagulation. Tsujikawa *et al*.[3] reported that such remote lesions have only a minor effect on the visual outcome.

With respect to the second comment “it can be difficult to treat polyps in the peripapillary area with PDT,”no eye had polyps around the disc in this study. Especially in Asians, the peripalliary PCV was reported to be rare as compared with macular PCV.[3]

In reply to the third comment, we know that large submacular hemorrhage due to PCV cannot be treated with PDT. We usually treat PCV with large submacular hemorrhage using intravitreal gas injection followed by PDT or IVB, and such cases were not included in this study.

With respect to the fourth comment, we know that repeated PDT may lead to choroidal damage. However, it has been reported that PDT combined with IVR in an animal model did not adversely affect the recanalization of the choriocapillaris as compared with PDT alone,[4] suggesting that choroidal damage due to PDT may be reduced by combining with it. Ruamviboonsuk *et al*.[5] reported that there was no permanent nonperfusion affecting choriocapillaris after the combination therapy of PDT and IVR. Sato *et al*.[6] reported that the combination therapy of PDT and IVB may reduce the retreatment rate and the occurrence of subsequent submacular hemorrhage as compared with PDT monotherapy. As for other adverse events, retinal pigment epithelial tear can occur not only after PDT but also after IVB.[7]

In reply to the comment “considering the economic burden associated with PDT,” continuous monthly IVR is more expensive than PDT. Most recently, it has been reported that combination therapy of PDT and IVR for PCV showed encouraging results concerning visual acuity (VA), incidence of subretinal hemorrhage, and recurrence of polyps.[5] VA improvement 6 months after IVR was reported to be 7.2 letters,[8] but VA improvement after combination therapy of PDT and IVR was 11.6 letters.[5] Kokame *et al*.[8] described that visual outcomes of IVR monotherapy for PCV may be worse than those for exudative age-related macular degeneration. Taken together, we disagree with the comment “anti-VEGF drugs alone could be the preferred treatment for symptomatic PCV.”
